# Corporate Sustainability Paradox Management: A Systematic Review and Future Agenda

**DOI:** 10.3389/fpsyg.2020.579272

**Published:** 2020-11-20

**Authors:** Ben Nanfeng Luo, Ying Tang, Erica Wen Chen, Shiqi Li, Dongying Luo

**Affiliations:** School of Labor and Human Resources, Renmin University of China, Beijing, China

**Keywords:** corporate sustainability, paradox, tension, corporate social responsibility, social enterprise

## Abstract

Increasing evidence suggests that corporate sustainability is paradoxical in nature, as corporates and managers have to achieve economic, social, and environmental goals, simultaneously. While a paradox perspective has been broadly incorporated into sustainability research for more than a decade, it has resulted in limited improvement in our understanding of corporate sustainability paradox management. In this study, the authors conduct a systematic review of the literature of corporate sustainability paradox management by adopting the Smith–Lewis three-stage model of dynamic equilibrium. The results reveal the following: (1) Both environmental and cognitive factors manifest tensions arising from the sustainability paradox. (2) While both proactive and defensive strategies are adopted to manage the tensions embedded in the corporate sustainability, the proactive strategy is more extensively studied in the current literature. (3) Management strategies of corporate sustainability paradox are characterized as multi-level, multi-stage, and dealing with multiple paradoxes. (4) Proactive strategies enable organizations to enjoy short-term and long-term sustainability benefits. The authors call for further research explicitly addressing the following areas: (1) the paradoxical nature of corporate sustainability management; (2) corporate sustainability paradox management of for-profit organizations; (3) the micro-foundations of corporate sustainability paradox management; (4) defensive strategies and new proactive strategies; and (5) a unified standard of sustainability outcomes. The practical implications of this review are then elaborated. In practice, the results imply that organizations would best manage the corporate sustainability paradox by understanding the paradox and its equilibrium stages. This review and proposed research agenda are expected to deepen interdisciplinary knowledge and set the stage for interested scholars to undertake in their future inquiries.

## Introduction

Although organizations have been pursuing their economic goals as primary interests for a long time, concerns about the natural environment and social welfare have become essential for organizations to deal with when considering sustainable development (Gladwin et al., 1995; Bansal, 2002; Zollo et al., 2013). Corporate sustainability juxtaposes economic, social, and environmental goals in parallel (Bansal, 2005; Schaltegger et al., 2016) to achieve overall social welfare (Schwartz and Carroll, 2008). Organizations that proactively take up their social responsibility display impressive competitiveness and vitality (Thiel, 2017; Ivory and Brooks, 2018; Daddi et al., 2019). Meanwhile, socially or environmentally irresponsible behavior still commonly occurs, even though it hurts the companies' image and reputation (Lin-Hi and Müller, 2013). While it is challenging to engage simultaneously with economic, social, and environmental goals, it is critical to consider how organizations and managers can successfully manage these multiple goals is critical to gain long-term competitiveness.

Three theoretical perspectives have emerged to address the relationships among economic, social, and environmental responsibilities: business frame, win–win, and business case perspectives. The business frame perspective from earlier scholarly work proposes that pursuing profit goals is an organization's primary responsibility and criticizes other pursuits as insignificant, as these essentially contradict an organization's economic benefit (Friedman, 1970). Corporate sustainability scholars have almost abandoned such a view, as it assumes a narrow scope of corporate responsibility and ignores the social and environmental aspects of corporations' operations (Wood, 1991; Hahn and Figge, 2011; Barnett, 2019). By contrast, the win–win perspective argues that both economic and social missions are attainable at the same time (Van der Byl and Slawinski, 2015). However, this view overemphasizes their reciprocity or interdependence and ignores the tensions from conflicts between these missions (Turban and Greening, 1997; Albinger and Freeman, 2000; Wagner, 2010), and the trade-offs between specific stakeholder groups are not always avoidable (Bridoux et al., 2016). Meanwhile, the business case frame regards these conflicts as being canceled out, by advocating the instrumental utilization of social pursuits to advance economic aims (Hahn et al., 2015; Hafenbrädl and Waeger, 2017); however, the business case frame has been criticized as insufficient to depict the true notion of corporate sustainability (Dyllick and Hockerts, 2002; Hahn and Figge, 2011), resulting in opportunism and a lack of intrinsic motivation for corporate social responsibility (CSR) engagement (Nijhof and Jeurissen, 2010).

In this study, the authors attempt to adopt a fourth perspective, the paradox perspective, to review how previous organizational sustainability research has deepened our knowledge, echoing Margolis and Walsh's (2003) call for intricate theorization. The paradox perspective, building on the recent advancement of organizational paradox theory, posits that organizations are rife with persistent, contradictory, yet mutually interdependent demands (Smith and Lewis, 2011). The paradox theory has been applied to explain diverse organizational phenomena, including leadership (e.g., Zhang et al., 2015, 2017) and innovation (e.g., Andriopoulos and Lewis, 2009). For corporate sustainability, pursuing competing economic, social, and environment goals at the same time is essentially an organizational paradox (Hahn et al., 2014). The paradox perspective acknowledges the tensions underlying opposing goals. However, in contrast to other perspectives, the paradox perspective centers on the tensions with which organization and management scholars have little choice but to engage (Lindgreen and Maon, 2019), exploring how organizations and decision-makers experience and manage the tensions underlying the sustainability paradox (Margolis and Walsh, 2003; Gao and Bansal, 2013; Hahn et al., 2015). For example, in contrast to ignoring or eliminating conflicts (an “either/or” solution), scholars have found that embracing the tensions can lead to an innovative (“both-and”) solution, followed by sustainable outcomes (Tracey et al., 2011; Jarzabkowski et al., 2013; Jay, 2013; Van der Byl and Slawinski, 2015; Ortiz-de-Mandojana and Bansal, 2016).

In this study, the authors adopt the dynamic equilibrium model proposed by Smith and Lewis (2011) on organizational paradox theory, attempting to offer a systematic review of academic developments in the management of the corporate sustainability paradox. The dynamic equilibrium model is influential in the literature of organizational paradox. It assumes that opposing forces of a paradox are dynamic rather than static, are persistent rather than transient within complex systems, and can be beneficial and powerful rather than detrimental and threatening. Specifically, the model encompasses three stages: how latent tensions turn into salience, how management strategies enable reinforcing cycles, and the outcomes of paradox management. The first stage introduces environmental factors and individuals' cognition and rhetoric as the major two types of manifestation forces that render the latent paradoxical forces (e.g., social goal and economic goal) salient and tensional. The second stage proposes how resolution and acceptance strategies fuel vicious and virtuous cycles. The last stage describes how, while focusing on one goal might achieve short-term success, long-term sustainability depends on constant efforts to address the diverging goals in a mutually reinforcing fashion. As a meta-theoretical perspective, the model encompasses the nature, approach, and impact of paradox management, providing a holistic guide to frame and explore how sustainability as disciplinary knowledge deals with its own inherent paradoxes (Smith and Lewis, 2011, p. 297).

The current work is not the first review on the paradox perspective in the corporate sustainability field. In an earlier review, Van der Byl and Slawinski (2015) identify four major approaches in corporate sustainability, that is, win–win, trade-off, integrative, and paradox perspectives. The reviewers mainly offer a conceptual distinction of the paradox perspective from the other three perspectives and highlight the emergence of the paradox perspective in corporate sustainability research. However, up to 2014, the year before Van der Byl and Slawinski's review was published, only eight articles (e.g., Berger et al., 2007; Wijen and Ansari, 2007) were found to adopt such a paradox lens. Later, in 2018, the *Journal of Business Ethics* published a thematic symposium on “paradoxes in corporate sustainability.” Among the issued articles, Hahn et al. (2018) are the first to develop a three-aspect framework, namely, descriptive, instrumental, and normative, and locate the six articles in the symposium within different aspects. Thereafter, the paradox perspective gained increasing recognition and sparked intensive research work among corporate sustainability scholars (e.g., Smith and Besharov, 2019; Hengst et al., 2020; Soderstrom and Heinze, 2020). However, as a disciplinary approach, this perspective is still in its infancy in the corporate sustainability area. Considering the rapid emergence of recent literature and the insufficient exploitation of existing research, a comprehensive and systemic review of corporate sustainability from the paradox perspective is timely and imperative. There is no preexisting published review specifically on this topic.

Guided by the three-stage dynamic equilibrium model, in this review, the authors aim to reveal the existing approaches to the manifestation of corporate sustainability tensions, the management of such tensions, and the downstream outcomes. In the rest of this article, the authors first outline the method for the selection of papers in the literature, then present the findings and discuss of a future agenda for further corporate sustainability research, and finally outline the implications and conclusions of the review.

## Research Methods

To conduct a systematic review of the literature of corporate sustainability from an organizational paradox perspective (Van der Byl and Slawinski, 2015), the authors followed the PRISMA guidelines (Moher et al., 2009) to the extent that they apply to non-medical systematic reviews. The time span for the search was up to December 31, 2019. To ensure the initial search was broad enough, the authors mainly followed two influential reviews, the CSR review of Aguinis and Glavas (2012) and the corporate sustainability review of Van der Byl and Slawinski (2015), and targeted a total of 35 top management journals and specialized niche ones (see Table 1). Finally, the authors searched in corresponding journal databases (e.g., Web of Science) or homepages, using the following keywords in the titles, keywords, or abstracts of the journal articles:

Paradox OR ambivalen^*^ OR ambidexterity OR integrat^*^ OR institutional logic OR tension OR dilemma OR conflict OR dialect OR Yin-yang OR Tao

AND

sustainab^*^ OR CSR OR social enterprise OR social entrepreneurship OR social partnership OR cross-section partnership OR hybrid organization OR microfinance OR social responsib^*^ OR social performance OR social responsiveness OR corporate citizenship OR corporate responsibility OR stakeholder responsibilities OR triple bottom line.

**Table 1:** Target journals for article collection.

**17 top management journals**	**4 specialized sustainability journals**	**14 journals selected based on the authors' experience and other concerns**
**Academy of Management Journal**, **Academy of Management Review**, **Administrative Science Quarterly**, International Journal of Management Reviews, Journal of the Academy of Marketing Science, Journal of Applied Psychology, Journal of International Business Studies, **Journal of Management**, **Journal of Management Studies**, Journal of Marketing, Journal of Occupational and Organizational Psychology, Journal of Organizational Behavior, Organizational Behavior and Human Decision Processes, **Organization Science**, **Organization Studies**, Personnel Psychology, **Strategic Management Journal** (Aguinis and Glavas, 2012; Van der Byl and Slawinski, 2015)	**Business and Society**, **Journal of Business Ethics**, Business Ethics Quarterly, Business Strategy and the Environment (Aguinis and Glavas, 2012; Van der Byl and Slawinski, 2015)	Other superior management journals: Academy of Management Annals, Academy of Management Perspectives, Annual Review of Organization Psychology and Organization Behavior, Leadership Quarterly, Human Resource Management, Management Science Other niche journals: Organization and Environment, Business Ethics: A European Review, Business and Society Review, Corporate Social Responsibility and Environmental Management, Management and Organization Review, Asia Pacific Journal of Management Practitioner journals*:* California Management Review, Harvard Business Review. (Van der Byl and Slawinski, 2015)

*Journals in boldface were mentioned in both reviews of Aguinis and Glavas (2012) and Van der Byl and Slawinski (2015)*.

**Figure 1:**
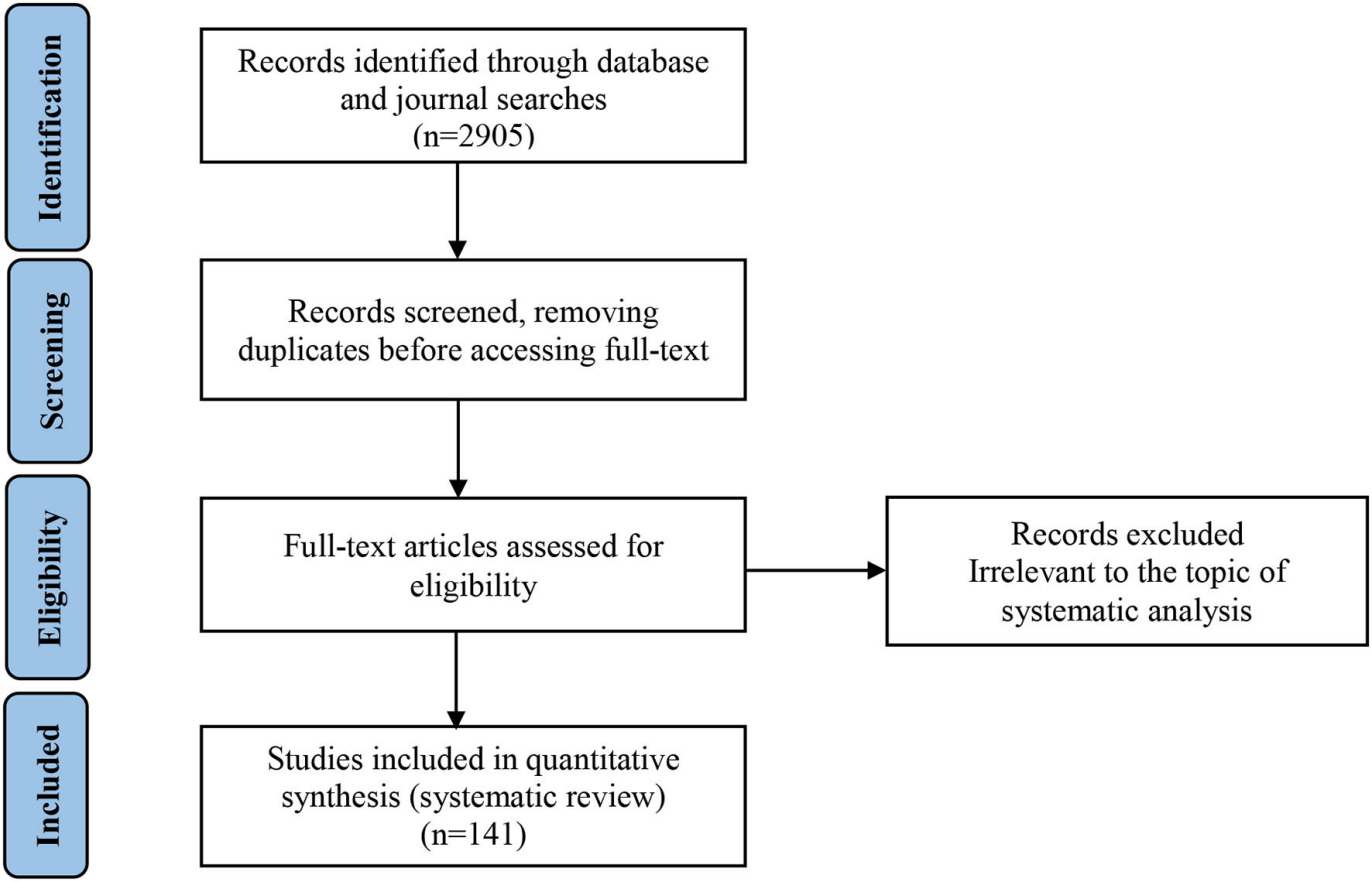
PRISMA 2009 flow diagram.

To reduce the risk of bias, the whole process of identification and selection of articles was guided by the PRISMA method (see Figure 1 for the PRISMA flow diagram). After searching for each pair of keywords, the initial search returned 2,905 articles, including repeated ones. After filtering out any articles repeated more than once, three authors (coders A, B, and C) scanned the abstracts of the remaining papers to rule out ones irrelevant, or with only minimum relevance. Each article was assigned to two coders. The expected agreement due to chance between coders A and B was 0.59 (κ = 0.96), that between B and C was 0.50 (κ = 0.98), and that between A and C was 0.53 (κ = 0.92). These results illustrate high inter-rater reliability. The articles obviously deflecting from the corporate sustainability theme were removed from the literature pool (these papers concerned business school curricular development, organizational learning, incentive systems, work meaningfulness, trust, organizational innovation, entry decision-making, risk management, supply chain management, etc.). For the remaining articles, the authors further skimmed through the full text to decide whether they should be included in the review and then cross-validated the results. In this step, articles were excluded mainly if they (1) did not reflect a paradox perspective of corporate sustainability; (2) merely focused on the business frame, business case, or win–win perspective, rather than the paradox perspective of corporate sustainability; and (3) had a main theme concerned with sustainability over and above the corporate level, such as government procurement (Preuss, 2007) and the rise of socially responsible investment funds at the regional or national level (Yan et al., 2019).

After the screening, the authors retained a total of 141 articles. Through this process, the extensiveness and concentration of the literature review were simultaneously guaranteed. The journal distribution of these articles is presented in Table 2.

**Table 2:** Distributions of articles in journals.

**Journals**	**Number of articles**
Journal of Business Ethics	59
Academy of Management Journal	10
Academy of Management Review	8
Business Ethics: A European Review	7
Organization Studies	7
Organization and Environment	6
Corporate Social Responsibility and Environmental Management	5
Business Ethics Quarterly	4
Journal of Management Studies	4
Business Strategy and the Environment	4
Administrative Science Quarterly	3
Journal of Organizational Behavior	3
Journal of the Academy of Marketing Science	3
Management and Organization Review	3
Organization Science	3
Strategic Management Journal	3
Asia Pacific Journal of Management	2
Journal of International Business Studies	2
Business and Society	2
California Management Review	1
International Journal of Management Reviews	1
Harvard Business Review	1
Total	141

When conducting further analysis, the authors mainly concentrated on the three stages that Smith and Lewis (2011) proposed in their study on the dynamic equilibrium model of organizational paradox. In this theoretical piece, building on earlier foundational work (e.g., Cameron and Quinn, 1988; Lewis, 2000), they define a paradox as consisting of “contradictory yet interrelated elements that exist simultaneously and persist over time” (p. 386). A paradox, by definition, cannot be eliminated. Furthermore, any attempt to eliminate the paradoxes will only spark more tensions, resulting in vicious cycles (Vince and Broussine, 1996). To holistically address this cyclical nature of experiencing and managing paradox, Smith and Lewis (2011) theorize three phases: how tensions arise from latency and turn into salience under the stimulation of environmental and individual factors, how actors take purposeful responses to manage paradox mainly through resolution and acceptance, and how short-term outcomes fuel long-term success. An increasing number of studies in broader organizational context have demonstrated that if actors can embrace and be more comfortable with the paradoxes rather than defending themselves and denying the existence of paradoxes (Miron-Spektor et al., 2018), a virtuous cycle would occur and eventually lead to sustainable development. Leaders who display paradoxical traits (e.g., humility and narcissism, Zhang et al., 2017) and are more prominent in leading their subordinates also enable work-promoted outcomes (Zhang et al., 2015). Thus, corporate sustainability studies focusing on addressing the cyclical nature of paradoxes and relevant management practices at different stages are needed.

The three stages and specific items that the authors focused on when this model was applied to investigate corporate sustainability issues are as follows: (1) the manifestation of paradoxical tensions, (2) the management of sustainability paradoxes, and (3) outcomes. First, the authors identified the factors that could render paradoxical tensions salient and categorize them into *environmental* and *individual* ones (i.e., environmental plurality, change, scarcity, and paradoxical cognition, respectively, according to the dynamic equilibrium model). Second, to depict the characteristics of these management processes, the authors analyzed the strategies and decided whether a *proactive* or *defensive* response is taken, and further coded these strategies as *multi-level* (e.g., involving managers from different levels), *multi-stage* (e.g., a dynamic and cyclic management process), or *multi-paradox* (e.g., both performing and organizing paradoxes are concerned). Finally, the authors investigated the *short-term impacts* (e.g., creativity and organizational learning) and *long-term outcomes* (e.g., economic and social performance enhancement) of these management strategies and their related practices. As for the initial screening of the focal articles, three of the authors independently analyzed the data. After each round of analysis, the authors discussed the coding together to resolve inconsistencies and ensure the accuracy and validity of the findings.

## Findings

In this section, the authors answer the following questions. First, what factors make sustainability tensions salient? Second, what are the strategies (e.g., proactive or defensive characteristics) to manage sustainability paradoxes? Third, what are the outcomes (short-term and long-term)? A list of the representative articles and their coding information is found in Table 3.

**Table 3:** Three-stage model of representative articles.

**Representative article**	**Activation**	**Strategies**	**Outcomes**	**Organizational type**	**Country**	**Method and time span**	**Level of analysis**	**Dynamic view**
Smets et al. (2015)	(Envi.) Plurality-multiple logics	Proactive	Tension mitigation in daily work	Business corporation (financial industry)	United Kingdom	Single-case study (180 days)	Individual level (frontline employees)	Yes
Acquier et al. (2018)		Proactive	Integrated CSR	Multinational business corporation (sports apparel industry)	Japanese headquarter and European subsidiaries	Single-case study (December 2013-July 2014)	Organizational and individual (managers) levels	Yes
Stadtler (2018)		Proactive	Advance on social agenda and align with the companies' individual goals	Cross-sector social partnerships	Egypt and Jordan	Comparative case study (August 2009 to September 2011)	Cross-organizational level	No
Davies and Doherty (2019)	(Envi.) Scarcity, complexity, and dynamics	Proactive	Automatic and contingent value spill-over	Social business	United Kingdom	Single-case study (17 years)	Organizational level	Yes
Smith and Besharov (2019)		Proactive	Increased revenues and social impacts	Social enterprise	Cambodia	Single-case study (2001-2010)	Organizational and individual (top managers) levels	Yes
Bruneel et al. (2020)	(Envi.) Plurality-multiple logics	Proactive	Sustainable hybrid governance structure and functioning	Social enterprise	N/A	Multiple-case study	Organizational level	Yes
Gümüsay et al. (2020)	(Envi.) Plurality-multiple logics (Cog.) Business frame vs. business case frame vs. paradoxical frame	Proactive	Less conflict-prone and more resilient	Hybrid organization (an Islamic bank)	Germany	Single-case study (24 months)	Organizational and individual levels	Yes
Schneider and Clauß (2020)		Proactive	Sustainable value and credibility for future value creation	Business corporation	Headquarter in Austria and Germany	Multiple-case study	Organizational level	No
Winkler et al. (2020)^*^	(Envi.) Change (Cog.) Attention, scrutiny, and interpretations	Proactive	Working through tensions	N/A	N/A	N/A	Individual level (managers)	Yes
Slawinski et al. (2020)		Proactive	Increased community well-being and enhanced built and natural environment	Regenerative organization	Canada	Single-case study (early 2012 to the end of 2017)	Organizational level	Yes
Soderstrom and Heinze (2020)	(Envi.) Plurality-multiple goals	Proactive	Sustainable practices	Food entrepreneurs and business collective organization	United States	Single-case study (May 2013 to January 2015)	Levels of business collective and entrepreneurs	Yes
Iivonen (2018)	(Envi.) Plurality-multiple demands	Defensive	Creation of outside secondary contradictions	Business corporation (beverage industry)	United States	Single-case study (2012–2014)	Organizational level	Yes
Ferns et al. (2019)		Defensive	Symbolic effect, little engagement in substantial climate change mitigation	Business corporation (oil and gas companies)	Mainly from Europe	Multiple-case study (1997–2015)	Organizational level	No
Sharma and Jaiswal (2018)	(Envi.) Change	Proactive vs. defensive	Profit or loss	Business corporation (a global pharmaceutical company)	India	Single-case study (5 years)	Organizational level	Yes
Daddi et al. (2019)		Proactive vs. defensive		Business corporation (paper production, textile/clothing, and leather)	Italy	Multiple-case study	Organizational level	No
Garst et al. (2020)		Proactive vs. defensive	Different levels of value sustainability	Business corporation (food industry)	Netherlands	Multiple-case study (2006–2016)	Organizational level	No

“*Envi.” denotes environmental factors; “Cog.” denotes cognitive factors*.

* *indicates the focal article to be theoretical*.

In corporate sustainability research (see Table 3), hybrid organizations, such as social enterprises, attract much attention. Other hybrid forms, such as cross-sector collaboration, are also discussed in sustainability studies. Unlike social enterprises, which focus on the centrality of their social mission (Chell, 2007), for-profit firms are under increasing attention because of their greater emphasis on economic values; nevertheless, this is still under-researched. On the methodology front, while single-case longitudinal research is still the most popular, quantitative examinations have been increasing in recent years.

### The Manifestation of Corporate Sustainability Paradoxes

Two sets of triggering factors that could manifest organizational paradoxical tensions have emerged, consistent with Smith and Lewis' (2011) dynamic equilibrium model, namely, environmental factors and actors' paradoxical cognition. The former emphasizes plurality, change, and the scarcity of the external environment, which all contribute to providing the material conditions of a paradox. The latter focuses on the actors themselves and highlights the role of personal frames and cognition. The research into paradoxical corporate sustainability also underlines these factors (see Table 3).

#### Environmental Factors

In general, organizational paradox research has recognized the importance of environmental factors in providing the material conditions that lead to perplexing choices (Scherer et al., 2013). In the context of accelerated globalization (Slawinski et al., 2020), marketization and professionalization (Bruneel et al., 2020), environmental degradation (Daddi et al., 2019), and public health concerns (Iivonen, 2018), plurality, change, and scarcity emerging from complex environments enable sustainability paradoxes to occur, owing to competing economic, social, and environment goals (Soderstrom and Heinze, 2020). Specifically, plurality has been manifested in the pursuit of the triple bottom line (Elkington, 1997), multiple institutional logics (Dahlmann and Grosvold, 2017), and multiple stakeholder demands (Smith et al., 2013). Any excessive concern about any goal may be detrimental to sustainability outcomes (Jay et al., 2017; Soderstrom and Heinze, 2020); thus, tensions appear. These kinds of tensions would directly reflect on firms' mission statements that combine economic pursuits and social or environmental visions. Some researchers show that the co-existence of social and economic/financial logics (Yan et al., 2019; Bruneel et al., 2020), market and community logics (Smets et al., 2015), or separate and integrated logics (Gümüsay et al., 2020) would provoke thorough tensions around the organization's structure and identity and change would ensue as institutional logics define the material practices, assumptions, values, and beliefs of organizations (Thornton et al., 2012). Other researchers, mainly from the perspective of stakeholder management, find that tensions would emerge as a consequence of heterogeneous opinions, confusion around roles, and diffused power or responsibility (Slade Shantz et al., 2020) when considering a wider range of stakeholders (both insiders and outsiders) in the sustainability context, of whom there are essentially inconsistent expectations (Iivonen, 2018).

Many studies have stressed that, apart from changes due to prominent trends, the dynamic nature of an organization's environment requires proactive adaptation for both the present and the future (Waldman and Bowen, 2016). For instance, the changes in organizational temporal structure and leadership styles (Sharma and Jaiswal, 2018), which are deeply rooted in a disparity in practice between “what should be” and “what is” (Winkler et al., 2020), would significantly provoke corporate sustainability tensions.

The triggering role of scarcity is magnified when researchers investigate special settings, such as entrepreneurship and social enterprises. Entrepreneurs with a paradoxical frame have limited resources, time, and expertise for sustainability options (Hahn et al., 2014). Davies and Doherty (2019) suggest that social enterprises struggle with more intensive sustainability tensions because of their insufficient attractiveness for capital, the difficulty of hiring employees with similar identities, and stakeholder legitimacy.

However, almost the entire discussion around environmental factors concentrates at the theoretical level. At this conceptual level, contradictory elements manifest more than interrelated ones, which essentially distinguishes paradoxical tensions of corporate sustainability from other tensions.

#### Actors' Paradoxical Cognition

Besides environmental factors, individuals' cognition is found to play an important role in manifesting sustainability tensions. Some researchers consider that perceived uncertainty, complexity, and ambiguity would add to individuals' cognitive burden (Sharma, 2000; Hahn et al., 2014), while others regard ambiguity as an important resource of identification (Eisenberg, 1984), allowing dissent among situated understandings to appear (Carollo and Guerci, 2018; Sharma and Jaiswal, 2018). In general, scholars agree on the idea that personal framing triggers the salience of the corporate sustainability paradox and paradoxical framing is the key to such transformation. For example, Hahn et al. (2014) suggest that, with a paradoxical framing, individuals would show higher sensitivity to sustainability tensions, which encourages individuals' acknowledgment of the interdependence and contradictions among their organization's competing goals (Vallaster et al., 2020). However, Child (2020, p. 1148) reveals that practitioners in the social enterprises could frame away the potential paradox by mechanisms such as “looking at the big picture,” “engaging with potentially paradoxical conditions rather than turning from them,” and “making favorable comparisons.”

Previous studies also advocate that the recognition of sustainability paradoxes can be a process, during which rhetoric operates as a main communication and sense-giving tool (Waldman and Bowen, 2016; Acquier et al., 2018). For example, Winkler et al. (2020) determine that self-persuasive CSR rhetoric would draw attention to any practices deviating from an organization's ambitious vision, resulting in tensions.

### Managing Sustainability Paradoxes

#### Proactive vs. Defensive Responses

While firms constantly experience sustainability paradoxes, how firms or critical actors respond varies. Broadly, responses or management strategies can be categorized as proactive versus defensive (Hahn et al., 2018; Iivonen, 2018). Three major distinctions have been made between the two types. First, active response requires acknowledging the existence of paradoxes and the correlation between their conflicting elements (Lewis and Smith, 2014), whereas a defensive response involves actors who often deny or ignore the existence of such contradictions. Second, proactively responding actors regard it as feasible to achieve multiple contradictory goals at the same time, whereas those with a defensive strategy tend to divert or eliminate the tension between economic, environmental, and social demands (Van der Byl and Slawinski, 2015). Third, actors adopting proactive responses often propose or make a fundamental change or a deviation from the existing stable state, while defensive responses constitute only a minor “natural extension” (Iivonen, 2018, p. 318) of the existing pattern.

##### Proactive strategies

Proactive strategies encompass two critical components, differentiation and integration (Poole and Van de Ven, 1989; Smith and Tushman, 2005; Andriopoulos and Lewis, 2009; Smith, 2014). Differentiation includes spatial and temporal separations of competing elements within different boundaries (Smith et al., 2013). For example, the segmentation mechanism divides partnerships into sub-tracks, which then act as intermediaries among cross-departmental partners to buffer conflicts (Stadtler, 2018). Moreover, by separating and differently managing stakeholders' benefits and expectations, a hybrid organization could successfully navigate between “community-focused” and “client-focused” orientations (Kannothra et al., 2018). For integration, previous work highlights the role of linking mechanisms (Hahn et al., 2016). A structural design, such as a hybrid set of members of the board (Bruneel et al., 2020), could combine business and social logics, and formal processes that regulate cross-function interaction (Battilana et al., 2015) could facilitate coordination and resource exchange. The authors observe that integration mechanisms are specifically addressed by some multinational enterprises. For example, Acquier et al. (2018) observe the international Japanese enterprise processes in multi-functional teams and cross-regional CSR projects to achieve highly integrated CSR, namely, increased professionalization and globalization, worldwide coordination, and the institutionalization of CSR. Some researchers argue that both differentiation and integration are indispensable for the organization (Smith and Tushman, 2005; Smith and Lewis, 2011) and can be utilized ambidextrously to produce a synergistic effect. For instance, Smets et al. (2015) came up with a segmenting–bridging–demarcating loop whereby both integration and differentiation are applied to daily work. When entering the German market, an Islamic bank uses polyphony (segmentation) to support individuals' practices and polysemy (integration) to provide uniformity, which combine to foster organizational elasticity (Gümüsay et al., 2020). However, it is noteworthy that the current literature fails to answer the extent to which the firm should differentiate between and integrate its competing economic, social, and environmental goals simultaneously.

Existing research has investigated other proactive paradox management practices. First, well-utilized communication, as is important in the CSR research (e.g., Zhao et al., 2020), could be a vital method to engage in the sustainability paradox. A good example could be the transformation from self-persuasive rhetoric to the three-step agonistic CSR rhetoric (Winkler et al., 2020). By using (1) invitational rhetoric to define visions as provisional and revisable, (2) listening rhetoric to understand the reasons for dissension, and (3) rearticulation rhetoric to transfer authority from the speaker to the audience, the dynamic communication process finally leads to a virtuous cycle. Apart from internal conveyance, external communication strategies emphasizing the firms' commitment to the environment, which may be further reinforced by third-party certifications, could maintain customers' trust (Daddi et al., 2019).

Second, ongoing engagement of stakeholders helps them to live with paradoxes, as their expectations of conflicts are the primary source of paradoxical tensions. The interaction between managers and stakeholders could be regarded as a process of knowledge co-creation, generating the “power from below” to reshape the power relations to manage the tensions (Bolton and Landells, 2015), and the proactive engagement of both internal and external stakeholders could lead to mutual appreciation (Slawinski et al., 2020) to further address the paradoxical issues.

Third, some researchers regard paradoxical thinking itself as a valid approach to handle tensions, but the guardrails—leadership expertise, formal structures, stakeholder relationships—are still needed to keep the practice on track (Soderstrom and Heinze, 2020).

##### Defensive strategy

Existing research favors investigating the proactive kind of responses to the sustainability paradox. However, in practice, companies often adopt defensive actions (Lewis, 2000; Smith and Lewis, 2012). When an organization's primary goal is threatened (e.g., by institutional complexity), the organization tends to react defensively to minimize internal conflicts and/or external threats to its legitimacy. In the organizational paradox literature, a focus on defensive reactions to paradoxical situations has generated a vast array of categorizations (see Schad et al., 2016, for a recent review). For example, Lewis (2000) divides defensive reactions into six categories: splitting, projection, repression, regression, reaction formation, and ambivalence (Lewis, 2000; Iivonen, 2018). To blame a scapegoat, which is actually the combination of splitting and projection, and to simply repress unpleasant emotions and thoughts are much more heatedly discussed than other defensive reactions in the current corporate sustainability paradox management literature (Vince and Broussine, 1996). Personal framing, such as looking at a big picture or making favorable comparisons, could also be regarded as an effective defensive mechanism to neglect problematic elements (Child, 2020).

In addition, some researchers observe that firms adopt defensive actions to transfer tensions to their external stakeholders, such as customers. For example, Iivonen (2018) points out that the Coca-Cola Company completely denies the tensions between its economic goals and the obesity problem, blaming customers for their irrational choices and lack of self-regulation. Another example is the practice of the European Oil and Gas Supermajors, which reduce or avoid tensions through impression management (Ferns et al., 2019). By making use of three myths—the techno-fix, the Promethean oilman, and climate partnerships—these companies have successfully legitimized their environmentally harmful businesses. Daddi et al. (2019) also investigate how paper producers, textile companies, and tanneries tried to balance environmental engagement and competitiveness through defensive strategies. To obtain recycled raw material, the paper producers focused on improving their competitiveness through more significant technological inputs or improved selection management. For instance, to avoid such tension arising from paradoxes, a tannery that produced high-quality leather for luxury brands directly sold chrome (a material used to produce leather) recovered from the process to tanneries that produced inferior leather. In general, organizations tend to adopt defensive responses when their core business collides directly with sustainability development, as they consider these contradictions to be irreconcilable and threatening their survival (Iivonen, 2018; Daddi et al., 2019).

##### Factors spurring proactive actions

The academic community concurs that defensive reactions would create negative feedback loops (Smith and Lewis, 2011) and ethical hazards (Hall et al., 2007) in the long run. Some researchers have investigated the individual and environmental factors that could encourage a proactive response and promote a virtuous cycle. Several scholars have reiterated the role of individual factors, including cognitive and behavioral complexity and emotion regulation, in the dynamic equilibrium model, among others (Corner and Pavlovich, 2016; Hahn et al., 2016; Waldman and Bowen, 2016; Slawinski et al., 2020). Leaders' capabilities to maintain consistency in their behavior and emotions while navigating toward inconsistent goals are particularly highlighted (Smith and Lewis, 2011; Slawinski et al., 2020). Prior work has also pointed out some organizational factors in shaping the actions taken concerning the tensions. In addition to the organizational dynamic capability proposed in the dynamic equilibrium model (Smith and Lewis, 2011; Schneider and Clauß, 2020), strategic agility, including strategic sensitivity, collective commitment, and resource fluidity (Ivory and Brooks, 2018), and knowledge absorptive capability (Garst et al., 2020) have been found to be crucial in managing the corporate sustainability paradox.

#### Characteristics of Management Strategies

Corporate sustainability paradoxes are “not linear or singular but were experienced by any actor, at any stage in the strategy process, according to the specific tasks they were implementing” (Hengst et al., 2020, p. 258). When handling the sustainability paradox, changes in the external environments may trigger new tensions that are sustained in accordance with previous tensions (Hahn et al., 2018). Thus, to manage sustainability paradox, coordination at different levels and stages as well as varied types of paradoxes have to be considered.

##### Multi-Level

Tensions in sustainability can appear at different levels, including individual, organizational, and societal levels (Hahn et al., 2015). Most prior research concentrates on the individual and organizational levels. At the individual level, sustainability-related managers and key decision-makers, rather than employees, are the primary targets of focus. For instance, Carollo and Guerci (2018) explore how sustainability managers manage ambivalence in identity, and Wry and York (2017) analyze how social entrepreneurs understand the contradictions between different logics. At the organizational level, most studies investigate tensions in inter-organizational and cross-sectional (i.e., government, business, and civil society) relations (Le Ber and Branzei, 2010; Vurro et al., 2010; Stadtler, 2018). In mainstream CSR research, some efforts have been made to connect the micro-level to the macro-level (e.g., Jones et al., 2017; Poonamallee and Joy, 2018), but organizational paradox scholars point out that cross-level interactions around the sustainability paradox are underexplored (Schad et al., 2016), with a few exceptions. For example, Soderstrom and Heinze (2020) investigate a business collective from a social aggregation perspective. They reveal how such a type of organization serves as an intermediary based on how individual actions could converge into organizational responses, and how organizational practice influences individuals' reactions. Furthermore, managers from middle management and top management teams could change and reshape each other's frames, including business, business case, and paradoxical frames, among others (Sharma and Jaiswal, 2018). Managers may also be influenced by stakeholders and react differently, such as by adjusting priorities and constraining discretion responsibilities (Liu et al., 2015). Notwithstanding the growing literature on this topic, societal level and cross-level investigations are still scant.

##### Multi-Stage

It is critical to recognize that the sustainability paradox is embedded in “long-term, iterative, reflexive, adaptive and co-evolving processes” (Smith and Lewis, 2011, p. 23). While most scholars agree that paradox management is a dynamic multi-stage process, researchers' understanding of what dynamic means varies. Some scholars have argued that, as enterprises develop, the management goes through different periods that require stage-specific investigations, whereas other scholars emphasize a circular process. A good illustration of the former view is Puma's practice in managing sustainability paradoxes in different periods (Baumann-Pauly et al., 2016). Puma has gone through three main development stages, paired with specific communication strategies for each. When forming connections with stakeholders, Puma first used a universal language through a dialogue platform. Then, Puma tried to select an appropriate language and reasons for each stakeholder group establishing common ground. Finally, Puma adopted a mixed legalization policy. A new reporting structure that directly linked sustainability data to financial data was developed in the third period. While these conversations may initially have been manipulative, they resulted in committed partnerships over time.

The existing literature has proposed a cyclic approach to understanding the dynamic nature of the sustainability paradox. Amaeshi (2010) uses a case study to develop a recursive model of paradoxes in social enterprises, conceptualizing the governance paradox as part of a social behavior cycle consisting of social context and structure. The governing paradox requires a (re)interpretation of the paradox, leading to a (new) continuous cycle of action. The recursive model describes the cyclical process of gradually improving the ability of boards to understand and manage these paradoxes over time. Smets et al. (2015) explore how insurers balance the contradictory relationship between market logic and community logic. They find that only the segmentation practice needs to be bridged, that bridging leads to demarcating, and that demarcating is confirmed again as a method to minimize this contradiction and maintain logical clarity. Therefore, these three mechanisms follow a circular process. Slawinski et al. (2020) also present a management cycle of a social enterprise with the process of constantly confronting new tensions, reinterpreting the meaning of identity, and experimenting with new practices.

##### Multi-Paradox

Organizational paradox theorists have categorized organizational paradoxes into various types. For example, Feldman and Pentland (2003) depict the tensions in organizational routings as stability vs. flexibility, Waldman and Bowen (2016) identify the tensions between agency and communion in leadership practice, and Besharov (2014) argues that there are relational tensions between managers and frontline employees. To understand the nature of paradoxes better, a more nuanced typology has been developed. Here, the authors keep with the widely accepted four-type categorization, namely, performing, learning, organizing, and belonging paradoxes, following Smith and Lewis (2011). While the sustainability paradox can be any combination of these four types of paradoxes, the authors contend that the performing type is most fundamental to understand the sustainability paradox. A performing paradox arises from competing goals, demands, and strategies, such as the simultaneous pursuit of economic, social, and environmental goals (Hahn et al., 2018). Most existing research on the sustainability paradox focuses on the performing paradox.

Some other researchers have also looked at multiple types of sustainability paradoxes. For example, the co-existence of a social mission and economic goals can involve various types of paradoxical tensions. First, an organizing paradox emerges when firms hire and socialize employees (Battilana and Dorado, 2010; Battilana et al., 2015), or (re)design their organizational structure (Battilana et al., 2012; Stadtler, 2018) and legal forms (Haigh and Hoffman, 2011; Battilana et al., 2012). Second, a belonging paradox occurs when organizational actors have opposing values, beliefs, or identities while confronting and resolving a set of competing goals (Phillips and Tracey, 2007; Battilana and Dorado, 2010; Smets et al., 2015; Demers and Gond, 2020). For example, employees conceptualize their roles differently in CSR activities (Seivwright and Unsworth, 2016). Third, a learning paradox could be salient since success in sustainability requires both short-term and long-term efforts (Midttun, 2007; Hahn, 2009; Pache and Santos, 2013).

However, in articles that identify multiple types of sustainability paradoxes, regardless of how the authors classify the types, they tend to simplify the complexity underlying the simultaneously co-existing paradoxes. Instead, the existing literature has concentrated on certain types of paradoxes (mostly the performing paradox), and/or discussed the identified paradoxes separately. This separation tendency could constrain our understanding of the sustainability paradox's intricate nature. A few exceptional studies have appeared recently. For example, Vallaster et al. (2020) adopt a comprehensive framework to investigate how dynamic capabilities affect various paradoxes differently. Winkler et al. (2020) show how agonistic rhetoric solves performing, organizing, and belonging paradoxes jointly. Therefore, the authors conclude that the complex relationships between various types of sustainability paradoxes, the interaction between paradoxes and management strategies, and the management process merit further research.

### Resulting Downstream Effects

Theoretically, proactive responses to organizational paradoxes could lead to sustainable outcomes. In the short term, individuals, teams, and organizations are likely to display excellence through enhanced learning and creativity, flexibility and resilience, and stimulated potential, which would pave the way for long-term success (Smith and Lewis, 2011).

#### Short-Term Effects

A body of research has empirically proved that proactive strategies could lead directly to tension mitigation. For instance, Smets et al. (2015) reveal how contradictory elements could co-exist, alleviating tensions and bringing benefits in daily work at the same time. The proactive shift toward agonistic rhetoric helps the organization to transcend tensions and to foster CSR (Winkler et al., 2020). Proactive strategies could also facilitate knowledge creation. Bolton and Landells (2015) suggest that the knowledge base created during the process of management–stakeholder interaction could further generate the power that impacts business vision. While Hahn et al. (2014) theoretically propose that sustainability challenges stimulate creative insights, Calic and Mosakowski (2016) prove that a sustainability orientation improves the innovation of entrepreneurs' final projects. Wry and York (2017) also show that entrepreneurs who navigate dual logics have greater possibility of proactively integrating these and developing creative business models. Gümüsay et al. (2020) regard this resilience as the ability the organization gains from the adoption of a proactive mechanism to avoid permanently suffering the loss or being estranged. This can help the corporation to perceive and correct a tendency toward deviation and to handle emergencies. Some researchers have discussed how the management of the corporate sustainability paradox affects corporate legitimacy. For example, sustainability efforts (Calic and Mosakowski, 2016) and paradoxical thinking (Scherer et al., 2013) could enhance and maintain the legitimacy gained from collective awareness and value (Dart, 2004). Moreover, organizations could obtain other instant benefits, such as corporate credibility (Schneider and Clauß, 2020), greater market engagement (Kannothra et al., 2018; Soderstrom and Heinze, 2020), and competitiveness (Fosfuri et al., 2016) by positively reacting to sustainability paradoxes, which would contribute to long-term value creation.

#### Long-Term Effects

Scholars have usually used synergistically increased organizational economic and social performance to depict sustainability success, even though economic and social pursuits could be detrimental to each other in the short run (Margolis and Walsh, 2003) and the effects on social or environmental performance are assumed only to manifest after a long period of time (Hoffman et al., 2010). Typical indicators of economic performance include profit, sales, or their annual rates of increase (Pache and Santos, 2013). Social performance is reflected in greater social impact, such as higher social employment (Pache and Santos, 2013; Battilana et al., 2015; Smith and Besharov, 2019), community regeneration (Slawinski et al., 2020), and community income increase (Kannothra et al., 2018). Proactive responses to the corporate sustainability paradox, which is the determining precondition for a virtuous cycle (Smith and Lewis, 2011), have been verified to enhance economic and social performance simultaneously (Crilly and Sloan, 2012; Pache and Santos, 2013; Smith and Besharov, 2019).

By contrast, defensive reactions that would lead to a vicious cycle cannot facilitate sustainable development (Smith and Lewis, 2011). Defensive responses, which are usually argued to be a potential barrier to sustainability, may counterintuitively bring some positive effects (Ferns et al., 2019). For example, a luxury leather producer that sells recycled material to other tanneries and refuse to produce inferior leather itself (a defensive strategy) successfully maintains the trust of its customers toward their own product quality (Daddi et al., 2019).

## Agenda for Future Research

In this study, using the PRISMA methodology, the authors reviewed the existing research with a paradox perspective on sustainability. By adopting Smith and Lewis' (2011) dynamic equilibrium model, the authors revealed how the prior sustainability literature concerns the investigation of the manifestation process of tensions among sustainability goals, the management strategies that organizations use to respond to the tensions, and resulting outcomes.

During synthesizing and mapping of previous studies, ample future research opportunities appear. First, the authors propose that the nature of the corporate sustainability paradox (i.e., its constitution vs. its social construction) requires further exploration, including theoretical clarity and empirical examination. In the mainstream organizational paradox literature, there is an ontological debate on whether paradoxical tensions are inherent in the system or socially constructed (Clegg, 2002; El-Sawad et al., 2004; Ashcraft et al., 2009). Smith and Lewis (2011) propose an integrative approach by arguing for the corporate sustainability paradox's dual nature in simultaneously highlighting the roles of material conditions and organizational actors during the manifestation process. With a quantum approach, Hahn and Knight (2020) conceptualize the paradox as both inherent and socially constructed (for a counterpoint, see Li, 2020).

This debate penetrates sustainability research and potentially shapes management strategies. With a few exceptions, such as Sharma and Bansal (2017), who directly bring the opposing poles together by revealing the interaction between action and cognition, the nature of the sustainability paradox in the current literature is yet to be fully explored. Rather than looking for clues as to whether the researcher holds a material view (e.g., Waldman and Bowen, 2016) or a socially constructive view (e.g., Child, 2020; Vallaster et al., 2020), this fundamental question requires more in-depth exploration.

Second, it is desirable to investigate the sustainability paradox management of for-profit organizations. When investigating management of the corporate sustainability paradox, the main focus of the existing literature is on social enterprises or non-profit organizations, not for-profit organizations (Smith et al., 2013; Battilana et al., 2015; Wry and York, 2017; Child, 2020). Yet tensions are also embedded in the often hybrid forms of for-profit organizations, bringing paradox management insights to researchers (e.g., Smets et al., 2015; Acquier et al., 2018; Schneider and Clauß, 2020). Moreover, for-profit organizations, originally holding a business logic, may experience hurdles that are significantly different from those of social enterprises when pursuing corporate sustainability. Therefore, drawing from the “tensions” between researching social organizations and for-profit business organizations, the authors suggest that an organization's form (non-profit vs. for-profit) could largely influence how the organization responds to and manages the sustainability paradox.

Third, the authors encourage more research on the micro-foundation of management of the corporate sustainability paradox. Organizational psychology would shed light on this line of research. There is a need to explore how the cognitions, attitudes, and emotions of managers and employees (cf., Chin et al., 2013; Li et al., 2020; Sarfraz et al., 2020) play a role in corporate sustainability paradox management. For instance, the significant role of paradoxical thinking is reflected in its dual effect. On one hand, individuals with the paradoxical frame are more acutely sensitized to and aware of the interconnections among seemingly opposing demands. On the other hand, a general tendency to endorse paradoxical thinking can “motivate” actors to seek solutions proactively. In the organizational paradox literature, paradoxical thinking has been identified as an effective factor to benefit creative behavior and in-role performance (Miron-Spektor et al., 2011, 2018). In the sustainability literature, some researchers have investigated the paradoxical frames of managers (Hahn et al., 2014; Sharma and Jaiswal, 2018; Schneider and Clauß, 2020), specifically CSR managers (Carollo and Guerci, 2018). The authors argue that paradox leadership (behavior) (Zhang et al., 2015, 2017) also has the potential to foster the paradoxical thinking and behaviors of subordinates, thereby creating a supportive climate for corporate sustainability.

Furthermore, the role of employee engagement together with its underlying micro-foundation are greatly ignored when using a paradox perspective compared to those in the traditional CSR research (Glavas, 2016; De Roeck and Maon, 2018). Based on the current efforts (e.g., Demers and Gond, 2020), the authors consider the mechanisms underlying the actors' cognitive frames to be missing in the context of sustainability issues. While Child (2020) mentions that one's cognitive frame can be altered, questions like how managers reconstruct their frames or how the manipulation of framings on suitability issues impact the paradox management and sustainability outcome would extend our understanding of the micro-foundation of the sustainability paradox (Sharma and Jaiswal, 2018).

Fourth, some researchers challenge the prevailing opinion on proactive and defensive strategies, which urges more investigations. Originally, a proactive strategy was favored as it would lead to a virtuous cycle and achieve sustainability outcomes (Smith and Lewis, 2011). There is increasing research, especially by those observing controversial industry practices, implying the lack of a direct link between proactive responses and immediately appealing performance outcomes (Iivonen, 2018; Ferns et al., 2019). Moreover, some recent studies have revealed successful sustainability achievement through defensive strategies, in addition to proactive management strategies (Daddi et al., 2019; Garst et al., 2020). The authors propose that these inconsistencies require further examination of hybrid of proactive and defensive strategies in corporate sustainability management.

Finally, a unified standard for a sustainability outcome is needed in future research. In some studies, the authors use the short-term effects to claim promising long-term achievements without substantial supporting evidence. In other cases, some researchers investigate whether the company could use its profit to cover the costs of social extension (e.g., Slawinski et al., 2020). Essentially, these examples reflect the lack of a unified standard on what sustainability outcomes actually mean. Therefore, the authors propose that sustainability scholars and practitioners should work together on a list of more effective indicators and a proper time span for which to evaluate sustainability outcomes.

## Discussion and Conclusion

The paradoxical nature of corporate sustainability, namely, the need to address social, ecological, and commercial concerns simultaneously, is at the core of corporate sustainability research. The literature on how corporations and firm decision-makers should address the sustainability paradox has rapidly evolved in the past decade. Hence, in this study, the authors reviewed existing sustainability research prior to 2020 when the paradox perspective received direct attention (Van der Byl and Slawinski, 2015; Hahn et al., 2018). After a systematic review of 141 articles published in the top management and corporate sustainability journals, the authors adopted Smith and Lewis' (2011) dynamic equilibrium model to inductively reveal how prior sustainability researchers have investigated how tensions have manifested among sustainability goals, the management strategies by which organizations respond to the tensions, and the resulting outcomes. The authors then offered a road map for future inquiries on integrating the organizational paradox perspective and corporate sustainability research.

This review has several practical implications. Leaders of firms would best manage the corporate sustainability paradox by understanding the paradox and its equilibrium stages. Understanding how and why firms are affected by opposing tensions and the stage they are in enables firms to better position their firms and develop their own strategies, for example, by pursuing social aims associated with their core business (Kaul and Luo, 2018). Even though firms struggle to reach all their competing goals, which constitutes the sustainability paradox (e.g., Davies and Doherty, 2019), the key to achieving sustainability is not to ignore or escape any contradictory goals. Instead, leaders should learn to be “paradox-savvy” to promote proactive strategies at different levels of their management practice (Waldman and Bowen, 2016).

For middle managers of firms, this review suggests that firms could provide development training on how middle managers attend to these tensions and how middle managers communicate this information from firm leaders and top managers to operational managers and employees. Middle managers are recognized as the major force resisting organizational change (Miles, 1997; Floyd and Lane, 2000). Without adequate commitment to organizational change actions, middle managers can cause organizational inertia or chaos (Huy, 2002). Therefore, attention should be paid to how middle managers, as the information synthesizer and facilitator between top and operating management levels, categorize, blend, and sell information on the sustainability paradoxes in the overall setting of a firm's strategies and implementations.

In conclusion, an understanding of how the existing literature has advanced knowledge of the management corporate sustainability paradox is timely for both scholars and practitioners alike. By incorporating Smith and Lewis' (2011) dynamic equilibrium model, this review has distinguished the paradox perspective from previous major approaches in corporate sustainability management. It further provides findings on how corporate sustainability tensions manifest and are managed, and how they impact firms and society as a whole.

## Data Availability Statement

The raw data supporting the conclusions of this article will be made available by the authors, without undue reservation.

## Author Contributions

BL proposed the research idea and developed the initial draft with YT. EC improved the theorizing. YT, SL, and DL collected, screened, and analyzed the articles together. BL, EC, and YT revised the draft. All authors contributed to the article and approved the submitted version.

## Conflict of Interest

The authors declare that the research was conducted in the absence of any commercial or financial relationships that could be construed as a potential conflict of interest.
